# Meta-analysis of SIRT1 expression as a prognostic marker for overall survival in gastrointestinal cancer

**DOI:** 10.18632/oncotarget.19880

**Published:** 2017-08-03

**Authors:** Shuangjie Wu, Jinghui Jiang, Jun Liu, Xinhai Wang, Yu Gan, Yifan Tang

**Affiliations:** ^1^ Department of General Surgery, Huashan Hospital, Fudan University, Shanghai 200040, China; ^2^ State Key Laboratory of Oncogenes and Related Genes, Shanghai Cancer Institute, Renji Hospital, Shanghai Jiao Tong University School of Medicine, Shanghai 200032, China

**Keywords:** SIRT1, gastrointestinal cancer, overall survival, prognosis, meta-analysis

## Abstract

Sirtuin 1 (SIRT1), a well-characterized NAD^+^-dependent histone deacetylase, is generally up-regulated in gastrointestinal cancers. However, the prognostic value of SIRT1 in gastrointestinal cancer remains inconclusive. Therefore, we report a meta-analysis of the association of SIRT1 expression with overall survival (OS) in gastrointestinal cancer. PubMed was systematically searched for studies evaluating the expression of SIRT1 and OS in patients with gastrointestinal cancer. Fifteen studies (six evaluating colorectal cancer, three evaluating hepatocellular carcinoma, three evaluating gastric cancer, and one each evaluating pancreatic cancer, esophageal squamous cell carcinoma, and gastroesophageal junction cancer) with 3,024 patients were finally included. The median percentage of gastrointestinal cancers with high SIRT1 expression was 52.5%. Overall analysis showed an association between high SIRT1 expression and worse OS [summary hazard ratio (sHR) 1.54, 95% confidence intervals (CI) 1.21-1.96] in gastrointestinal cancer. However, heterogeneity was observed across studies, which was mainly attributed to cancer type. Subgroup analysis revealed that SIRT1 was significantly associated with worse OS in non-colorectal gastrointestinal cancer (sHR 1.82, 95% CI 1.50-2.21), in particular in gastric cancer (sHR 3.19, 95% CI 1.97-5.16) and hepatocellular carcinoma (sHR 1.53, 95% CI 1.16-2.01), with no evidence of heterogeneity or bias. However, no association was observed in colorectal cancer (sHR 1.15, 95% CI 0.81-1.62). In conclusion, high SIRT1 expression is a potential marker for poor survival in non-colorectal gastrointestinal cancer, but not in colorectal cancer.

## INTRODUCTION

Cancers of the digestive system are among the most common types of malignant tumors worldwide [1]. Despite recent advances in the treatment of these malignancies, gastrointestinal cancers, especially liver, colorectal, and gastric cancers, remain responsible for a number of cancer-related deaths [1]. The search for novel molecular prognostic biomarkers in gastrointestinal cancers has been an ongoing task in recent decades. Epigenetics is a promising field for prognostic biomarker research, because aberrant epigenetic modification underlies not only the formation but also the progression of cancers. Numerous studies in this field have linked histone deacetylases, which constitute one class of epigenetic regulators, to clinical outcomes and prognoses in cancer patients [2].

Sirtuins, which are the mammalian orthologs of yeast silent information regulator 2 (SIR2), are NAD^+^-dependent histone deacetylases (class III histone deacetylases). Sirtuin 1 (SIRT1) is the most extensively characterized member of the sirtuin family, and has been reported to participate in various biological processes by the deacetylation of not only histones but also non-histone proteins [3]. Relative to tumorigenesis, the role of SIRT1 is equivocal. Although SIRT1 has been suggested to play a tumor-suppressive role [3], there has been convincing evidence arguing for its oncogenic properties. SIRT1 could deacetylate and suppress the function of several other tumor suppressors, such as p53 [4] and p73 [5], and increase the stability of the oncoprotein N-Myc [6]. In addition, SIRT1 has been shown to promote survival and inhibit apoptosis of cancer cells [7]. Recently, it was reported that transgenic SIRT1 expression promoted carcinogenesis in PTEN-deficient mice [8], whereas enterocyte-specific inactivation of SIRT1 reduced the tumor load in APC^+/min^ mice [9]. These data have further argued for an *in vivo* tumor-promoting function of SIRT1 during cancer progression.

The deregulation of SIRT1 expression has been found in various cancers [10]. For gastrointestinal cancers, the expression of SIRT1 is generally elevated [11–16]. A number of preclinical studies have suggested that blocking SIRT1 activity might be a promising strategy for various cancers of the digestive system [17–21]. However, although considerable attention has been focused on the prognostic significance of SIRT1, there was no conclusive evidence for its prognostic impact in gastrointestinal cancer. Regarding colorectal cancer, a couple of studies have reported significant associations between high SIRT1 expression and poor overall survival (OS) and/or disease-free survival [15, 16]. Associations of SIRT1 expression with poor survival have also been found in patients with other gastrointestinal cancers, including liver, pancreatic and gastric cancers [22–24]. However, several other reports either showed that there was no association between SIRT1 expression and survival outcome [25, 26] or found that the high tumoral expression of SIRT1 predicted better survival [27, 28].

Here, we present a meta-analysis that quantitatively summarized the existing evidence to evaluate the prognostic impact of SIRT1 expression on survival in gastrointestinal cancer. The aim of the current study was to estimate the role of SIRT1 in relation to OS in cancers of the digestive system.

## RESULTS

### Description of studies

**Figure 1:**
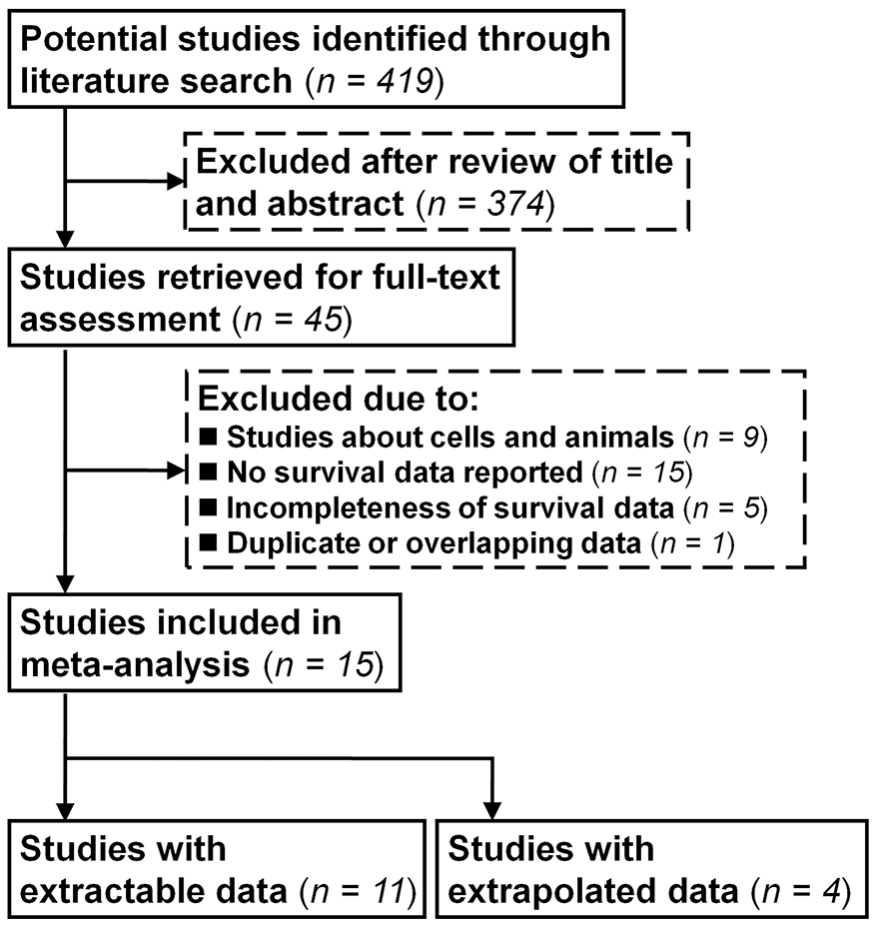
Flow diagram of study search and identification processes

As shown in the flow diagram of the study search (Figure 1), a total of 15 studies (3,024 patients) were finally included in the meta-analysis. The details of the included studies are shown in Table 1. Six studies evaluated colorectal cancer [15, 16, 25, 27–29], three evaluated hepatocellular carcinoma [12, 22, 26], three evaluated gastric cancer [24, 30, 31], and one each evaluated pancreatic cancer [23], esophageal squamous cell carcinoma [32], and gastroesophageal junction cancer [33]. Most of the included studies (12 of 15) were conducted in Asia [12, 15, 16, 22, 24, 26, 28-33], while the remaining studies were conducted in Europe (2 studies) [23, 27] and North America (1 study) [25].

**Table 1:** Main characteristics of included studies

Study	Year	Study size	Patient source	Tumor type	Follow-up	Stage	SIRT1 high expression (%)	Primary antibody used	Adjustiments	Quality score
Chen HC, et al	2012	172	Taiwan	HCC	Median, 10.4 years	I-III	55%	B10, Santa Cruz	Nil^a^	25
Lv L, et al	2014	120	China	Colorectal	Mean, 4.4 years	I-IV	NS	Clone E104, Abcam	Nil^a^	26
Chen X, et al	2014	102	China	Colorectal	NS	II-IV	43.1%	Clone E104, Abcam	Age, sex, metastasis, stage	24
Jang KY, et al	2012	154	Korea	HCC	Maximum, 11.6 years	I-IV	36%	H-300, Santa Cruz	Stage, AFP, albumin, c-Myc, p53	25
Stenzinger A, et al	2013	129	Germany	Pancreatic	Mean, 1.8 years	I-IV	27.9%	Clone E104, Epitomics	Stage, grade	25
Cha EJ, et al	2009	177	Korea	Gastric	Maximum, 10.4 years	I-IV	73%	H-300, Santa Cruz	Nil	27
Nosho K, et al	2009	485	United States	Colorectal	NS	I-IV	37%	Clone E104, Epitomics	Age, sex, year of diagnosis, tumor location, stage, grade, CIMP, MSI, KRAS, BRAF, PIK3CA, p53, β-catenin, FASN, COX-2, LINE-1 methylation	25
Benard A, et al	2015	254	Netherland	Colorectal	Mean, 8.6 years	I-III	50%	Clone E104, Abcam	Age, sex, stage, tumour location, tumour size, MSI	29
Jung W, et al	2013	349	Korea	Colorectal	Mean, 4.6 years	I-IV	67%	H-300, Santa Cruz	Age, tumor location, stage, grade, β-catenin	28
Jang SH, et al	2012	497	Korea	Colorectal	Mean, 5.9 years	I-IV	41.9%	B-7, Santa Cruz	Stage, grade	26
Hao C, et al	2014	99	China	HCC	NS	I-IV	76.8%	Clone E104, Abcam	Nil^a^	24
Zhang HL, et al	2013	176	China	GEJ	Maximum, 4.2 years	I-IV	46%	Clone E104, Epitomics	Nil^a^	28
Qiu G, et al	2016	96	China	Gastric	Mean, 31.6 months	I-IV	55.2%	H-300, Santa Cruz	Age, tumor size, grade, LN metastasis, tumor invasion, stage, Beclin1	30
Zhang S, et al	2016	45	China	Gastric	NS	I-IV	82.2%	Clone E104, Abcam	Sex, age, smoke, alcohol addiction, high blood pressure, T2D, HP infection	26
He Z, et al	2016	86	China	ESCC	NS	I-III	62.8%	Clone E104, Abcam	Age, tumor size, smoke, alcohol addiction, tumor location, stage, lymph node status, and differentiation	26

^a^Hazard ratio and variance were estimated based on Kaplan–Meier survival curve.

AFP, alpha-fetoprotein; NS, not stated; CIMP, CpG island methylator phenotyp; COX-2, cyclooxygenase-2; FASN, fatty acid synthase; MSI, microsatellite instability; LINE-1, long interspersed nuclear element-1; HCC, hepatocellular carcinoma; ESCC, esophageal squamous cell carcinoma; GEJ, gastroesophageal junction; T2D, type 2 diabetes; HP, helicobacterpylori.

### Evaluation and expression of SIRT1

All included studies used immunohistochemistry techniques for the assessment of SIRT1 expression. Although various antibodies were used for the evaluation of SIRT1 expression (Table 1), most of the included studies (9 of 15) used rabbit monoclonal anti-SIRT1 antibody E104 either from Epitomics [23, 25, 33] or from Abcam [15, 16, 26, 27, 31, 32]. Other studies used mouse monoclonal [12, 29] or rabbit polyclonal anti-SIRT1 antibody from Santa Cruz Biotechnology [22, 24, 28, 30]. Fourteen of the 15 studies reported the proportion of high SIRT1 expression [12, 16, 22-29, 33], and the median high expression of SIRT1 staining was 52.5%. The levels of high SIRT1 expression in colorectal cancer ranged from 37.0% to 67.0%, and ranged from 27.9% to 82.2% in non-colorectal gastrointestinal cancers.

### Overall analysis

**Figure 2:**
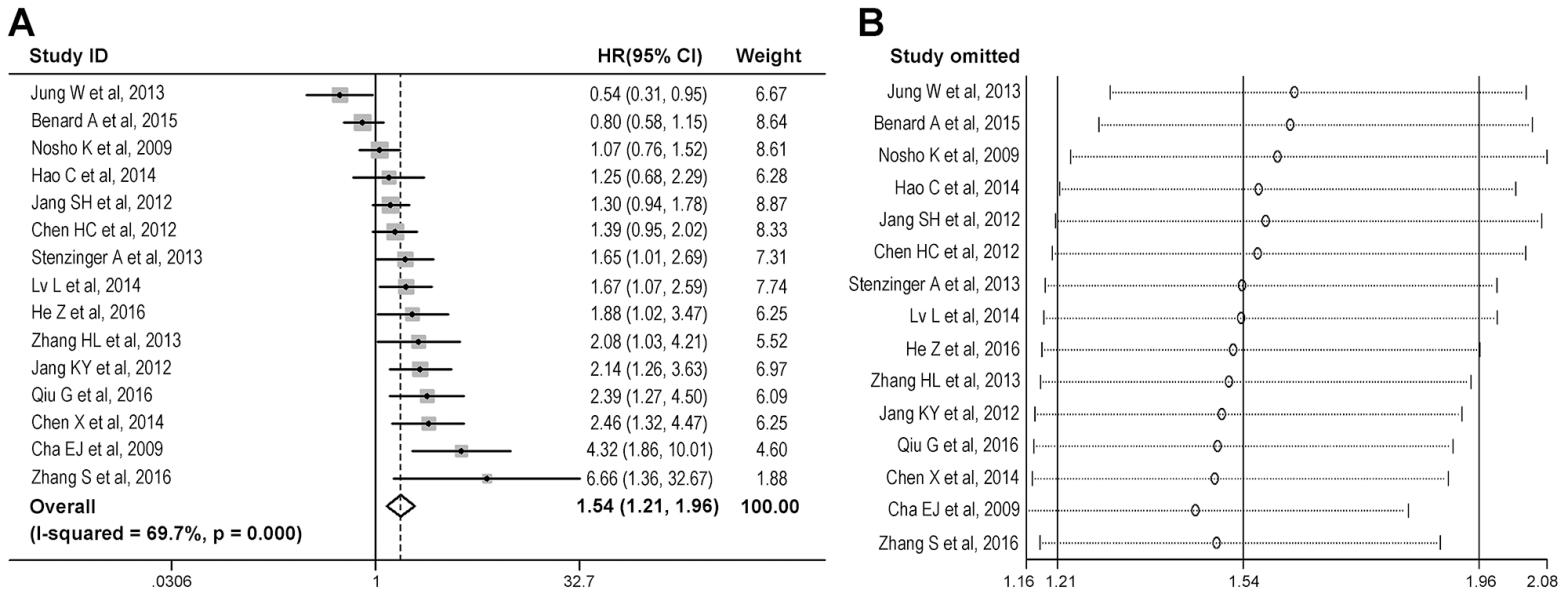
Overall analysis of the association between SIRT1 expression and overall survival in gastrointestinal cancer **(A)** Meta-analysis was conducted to estimate the summary hazard ratio of the association between SIRT1 expression and overall survival. **(B)** Sensitivity analysis was conducted to evaluate the influence of individual studies on the summary hazard ratio for overall survival.

The combined analysis of 15 studies showed that high SIRT1 expression was significantly associated with worse OS (Figure 2A), with a summary hazard ratio (sHR) of 1.54 [95% confidence interval (CI) = 1.21-1.96]. Significant heterogeneity was observed among studies [*I*^2^ = 69.7%, *P* value for heterogeneity (*P*_h_) < 0.001]. Sensitivity analysis which was conducted by omitting one study at a time from the pooled estimate, suggested that none of the individual studies substantially influenced the summary statistic (Figure 2B).

Potential sources of heterogeneity, including cancer type, geographic area, SIRT1 expression level, primary antibody used for the immunohistochemical evaluation of SIRT1 expression, method of data extraction, confounding adjustment, and quality score, were assessed with meta-regression analysis. As shown in Table 2, cancer type had a significant influence on the overall association between SIRT1 expression and OS (*P* = 0.037), suggesting that cancer type mainly contributed to the heterogeneity in the overall analysis. Several quantitative variables (year of publication, study size, and length of follow-up) were also included in the meta-regression analysis and were not found to be significant sources of heterogeneity.

**Table 2:** Meta-regression analysis of possible sources of heterogeneity

Possible source of heterogeneity	Residual *I*^2^	*P* value
Cancer type	60.22%	0.037
(colorectal cancer vs non-colorectal gastrointestinal cancer)		
Geographic area	65.38%	0.165
(Asia vs non-Asia)		
Percentage of high SIRT1 expression	72.08%	0.724
(≥53% vs < 53%)		
Primary antibody used for SIRT1 evaluation	72.19%	0.973
(rabbit monoclonal antibody E104 vs others)		
Method of data extraction	71.86%	0.976
(direct estimation vs indirect data extraction)		
Confounding adjustment	70.31%	0.474
(adjustment vs non-adjustment for confounding)		
Quality score	71.42%	0.863
(≤26 vs >26)		
Year of publication	71.99%	0.766
(per 1-year increment)		
Study size	77.34%	0.779
(per 100-patient increment)		
Length of follow-up	74.8%	0.443
(per 1-year increment)		

### Subgroup analysis

**Figure 3:**
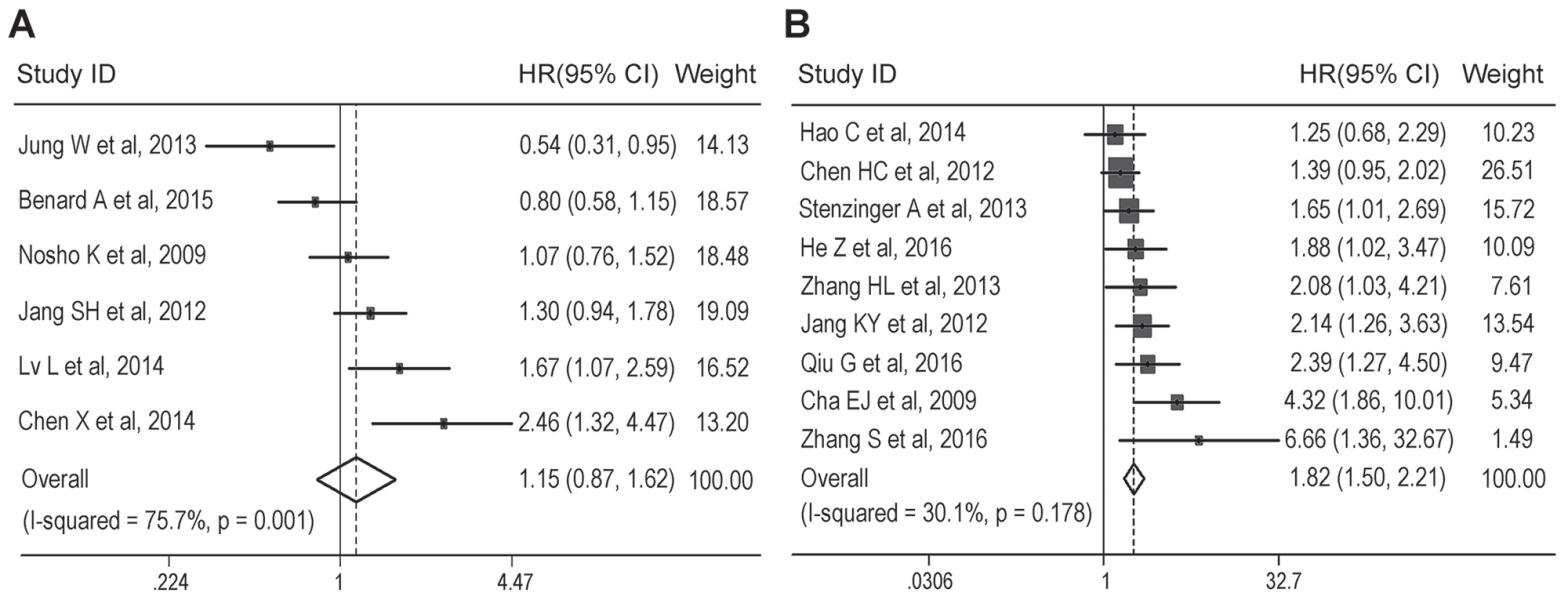
Subgroup analysis of the association between SIRT1 expression and overall survival according to cancer type Meta-analysis was conducted to estimate the summary hazard ratio (HR) of the association between SIRT1 expression and overall survival in patients with colorectal cancer **(A)** or non-colorectal gastrointestinal cancer **(B)**.

A subgroup analysis was first conducted according to cancer type. When we restricted the analysis to six colorectal cancer studies (1,807 patients) [15, 16, 25, 27–29], no association of SIRT1 expression with OS was evident, which resulted in a sHR of 1.15 with a 95% CI of 0.81-1.62 (Figure 3A and Table 3). Additionally, substantial heterogeneity was detected (*I*^2^ = 75.7%,*P*_h_ = 0.001). Another nine included studies analyzed the associations between SIRT1 expression and OS in hepatocellular carcinoma, pancreatic cancer, gastric cancer, esophageal squamous cell carcinoma, and gastroesophageal junction cancer [12, 22-24, 26, 30-33]. The combined analysis of these studies (1217 patients) showed that high SIRT1 expression was significantly associated with worse OS (sHR 1.82, 95% CI = 1.50-2.21) in non-colorectal gastrointestinal cancer, with no evidence for significant heterogeneity (*I*^2^ = 30.1%, *P*_h_ = 0.178) (Figure 3B and Table 3). For hepatocellular carcinoma (3 studies, 425 patients) and gastric cancer (3 studies, 318 patients), high SIRT1 expression was also significantly associated with worse OS (hepatocellular carcinoma: sHR 1.53, 95% CI = 1.16-2.01; gastric cancer: sHR 3.19, 95% CI = 1.97-5.16) with no heterogeneity (hepatocellular carcinoma: *I*^2^ = 10.9%, *P*_h_ = 0.326; gastric cancer: *I*^2^ = 5.7%, *P*_h_ = 0.346) (Table 3).

**Table 3:** Subgroup analysis of the association between high SIRT1 expression and overall survival in gastrointestinal cancer patients

Subgroup	No. of studies	sHR (95% CI)	*P*	*I*^2^ (%)	*P*_h_
Cancer type					
Colorectal cancer	6	1.15 (0.81-1.62)	0.443	75.7	0.001
Non-colorectal gastrointestinal cancer	9	1.82 (1.50-2.21)	<0.001	30.1	0.178
Hepatocellular carcinoma	3	1.53 (1.16-2.01)	0.002	10.9	0.326
Gastric cancer	3	3.19 (1.97-5.16)	<0.001	5.7	0.346
Geographic location					
Asia	12	1.72 (1.31-2.26)	<0.001	64.6	0.001
Asia / Colorectal cancer	4	1.30 (0.78-2.18)	0.318	79.7	0.002
Asia / Non-colorectal gastrointestinal cancer	8	1.86 (1.50-2.30)	<0.001	37.8	0.128
Non-Asia (Europe & North America)	3	1.09 (0.74-1.58)	0.671	64.9	0.058
Percentage of high SIRT1 expression^a^					
≥53%	7	1.72 (1.04-2.82)	0.034	76.3	<0.001
<53%	7	1.46 (1.12-1.91)	0.005	65.6	0.005
Primary antibody used for SIRT1 evaluation					
Rabbit monoclonal antibody E104	9	1.52 (1.13-2.04)	0.006	64.3	0.004
others	6	1.58 (1.02-2.45)	0.042	78.6	<0.001

^a^One included study did not provide the data about the levels of SIRT1 expression.

sHR, summary hazard ratio; CI, confidence interval; *P*_h_, *P* value for heterogeneity.

We also conducted subgroup analyses according to patients’ geographic location. As illustrated in Table 3, high SIRT1 expression conferred a significantly worse OS for gastrointestinal cancer patients from Asia (sHR 1.72, 95% CI = 1.31-2.26). However, no association was found in non-Asian patients (sHR 1.09, 95% CI = 0.74-1.58). A further subgroup analysis of Asian patients according to cancer type showed that there was a significant association between SIRT1 and worse OS in non-colorectal gastrointestinal cancer (sHR 1.86, 95% CI = 1.50-2.30), but not in colorectal cancer (sHR 1.30, 95% CI =0.78-2.18). Furthermore, similar results were obtained in the subgroup analysis according to the percentage of high SIRT1 expression (≥ 53%: sHR = 1.72, 95% CI 1.04-2.82, *P* = 0.034; < 53%: sHR = 1.46, 95% CI 1.12-1.91, *P* = 0.005). In addition, the combined analysis of the studies using the rabbit monoclonal anti-SIRT1 antibody E104 showed a significantly association of high SIRT1 expression with worse OS (sHR 1.52, 95% CI = 1.13-2.04). A similar result was observed when we included the studies that used other anti-SIRT1 antibodies for immunohistochemical analysis (sHR 1.58, 95% CI = 1.02-2.45).

### Publication bias

**Figure 4:**
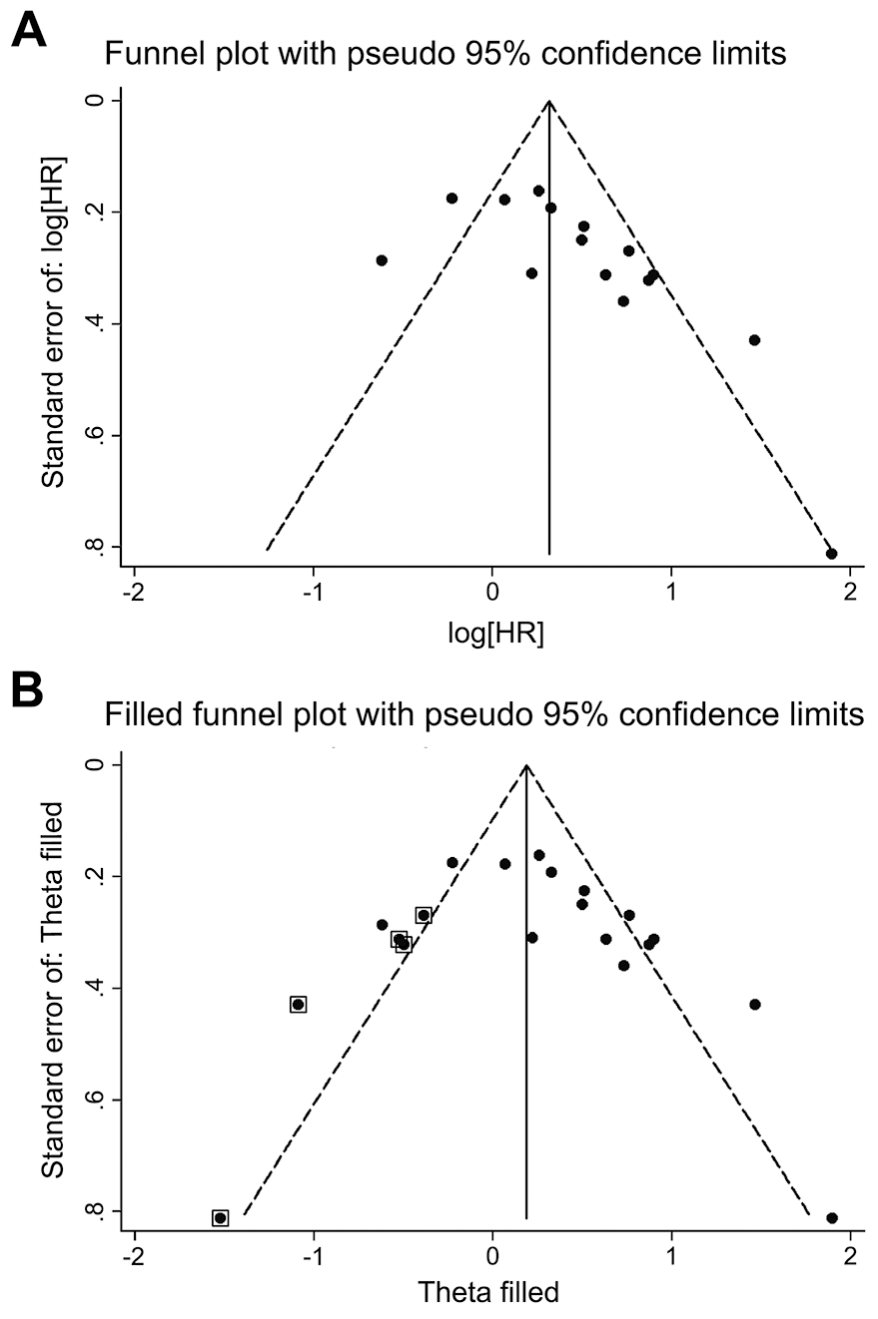
Funnel plot with pseudo 95% confidence limits **(A)** Funnel plot of the studies included in the meta-analysis of the association between SIRT1 expression and overall survival in patients with gastrointestinal cancer. **(B)** Filled funnel plot of all studies, including the five hypothetical studies, using the “trim-and-fill” method.

For the overall analysis of OS data, the funnel plot showed an asymmetric distribution (Figure 4A). Evidence of significant publication bias was detected by Begg’s test (*P* = 0.002), and by Egger’s test (*P* = 0.013). Non-parametric “trim-and-fill” method was utilized to estimating three missing studies (Figure 4B). After adjustment by “trim-and-fill” method, the estimated sHR was 1.38, with a 95% CI of 1.01-1.89.

## DISCUSSION

SIRT1 has been generally over-expressed in gastrointestinal cancers, including liver, pancreatic, and colorectal cancers [11–16], suggesting a putative role for SIRT1 consistent with tumor promotion. However, the prognostic value of SIRT1 in these cancers remains inconclusive. The current study meta-analyzed the published data about the expression of SIRT1 in gastrointestinal cancers and its associations with patients’ survival. All included studies assessed tumoral SIRT1 expression by immunohistochemistry. Furthermore, our results of meta-regression and subgroup analyses suggested that the primary antibody used for the evaluation of SIRT1 expression did not influence the summary estimate. Therefore, there was consistency in the assessment process among the studies.

The results of our overall analysis indicated a significant association of high SIRT1 expression with poor OS in gastrointestinal cancer, although the detected publication bias limited the conclusion that could be drawn. Also, it should be noted that considerable heterogeneity was observed in the overall analysis. The results of the meta-regression and subgroup analyses indicated that cancer type might be a major source of this heterogeneity. In non-colorectal gastrointestinal cancers, including hepatocellular carcinoma, pancreatic cancer, and gastric cancers, high expression of SIRT1 was clearly associated with worse OS, and there was no evidence of statistical heterogeneity or bias. Particularly, the result was consistent when we only included patients with hepatocellular carcinoma or gastric cancer. These results argued for a cancer-promoting function of SIRT1 during the oncogenic process in these cancers. There are several mechanisms involved in the oncogenic role of SIRT1. It has been documented that SIRT1 can establish and maintain silent chromatin via the deacetylation of histone proteins, thus protecting cells from apoptosis [34]. Additionally, SIRT1 can repress tumor suppressor genes, such as p53 and FOXO family members, either by directly binding and deacetylating these non-histone proteins or by inducing heritable CpG island methylation at the gene promoter [3, 34]. Studies focusing on the non-colorectal gastrointestinal cancers that were included in this meta-analysis have found that SIRT1 could promote chemotherapy resistance [12, 20, 35] and enhance invasive and metastatic potential by inducing epithelial-mesenchymal transition [26]. Inhibition of SIRT1 has been shown either to inhibit the growth of cancer cells or to reduce the tumor burden in animal models [17, 18, 21]. Additionally, blocking SIRT1 activity with specific inhibitors was suggested to reverse the chemoresistance of both gastric and pancreatic cancers [20, 35]. Taken together, the results from our analyses, together with previous findings, supported that SIRT1 is not only an adverse prognostic factor but also a potential therapeutic target, for non-colorectal gastrointestinal cancers.

In colorectal cancer, the overall evidence from published studies has been insufficient to establish a correlation between SIRT1 expression and OS. Our results supported the previous studies [25, 29], including a recent meta-analysis [36], showing that SIRT1 is not an independent prognostic factor for survival in colorectal cancer. However, the results should be interpreted with caution due to the significant heterogeneity among colorectal studies. This heterogeneity might be partly due to the variation in patient selection among the studies. For instance, one study included more patients with colon cancer and fewer patients with metastasis [27], whereas another study recruited fewer patients with colon cancer and more patients with metastasis [16]. Stratified analyses according to demographic or clinicopathological features (such as anatomic site and disease stage), which were not conducted in this meta-analysis due to the limited number of available studies, can be conducted in the future to further assess the prognostic value of SIRT1 in colorectal cancer when more primary studies are available.

Previous experimental studies using *in vitro* and *in vivo* models of human colorectal cancer have shown that SIRT1 exhibited pleiotropic effects, *i*.*e*., tumor-suppressive and growth-promoting effects, depending on cellular context [16, 37]. The heterogeneity observed in our combined analysis of colorectal studies was also possibly due to the complex role of SIRT1 in this particular cancer. However, the mechanisms involved in such different functions of SIRT1 in colorectal cancer remain unclear. Nevertheless, it is noteworthy that SIRT1 was specifically over-expressed in colorectal serrated lesions with KRAS or BRAF mutations, possibly contributing to their malignant transformation into colorectal cancer [38]. In addition, SIRT1 expression in colorectal cancer has also been associated with microsatellite instability and the CpG island methylator phenotype [25, 29], both of which have been linked to prognosis and survival in colorectal cancer [39, 40]. Further studies including SIRT1, as well as other molecular features, are required to assess its prognostic role more precisely.

In addition, our subgroup showed that high SIRT1 expression was significantly associated with worse OS in Asian patients, consistent with the result of our overall analysis. However, no association between SIRT1 and OS was observed in patients from non-Asian areas (including Europe and North America). These different associations may be due to the fact that most of the included studies (2/3) conducted in non-Asian areas evaluated colorectal cancer. The further stratified analysis of Asian patients according to cancer type showed a significant association of high SIRT1 expression with worse OS only in non-colorectal gastrointestinal cancer, supporting the results of the subgroup analysis including all patients regardless of geographic location. However, there was a problem of small sample sizes in the analysis of non-Asian patients. Further research is needed to investigate the prognostic role of SIRT1 in colorectal cancer and other gastrointestinal cancer in non-Asian areas.

This study had several limitations, which may affect the interpretation of some of our results. First, there was the problem of heterogeneity not only in the overall analysis but also in the subgroup analysis of colorectal cancer. Second, as we discussed earlier, SIRT1 expression has been associated with other molecular biomarkers for cancer prognosis. Inadequate adjustment for these molecular biomarkers in several included studies might have resulted in spurious associations, whereas the results of meta-regression analysis suggested that whether adjusting for confounding factors was undertaken was unlikely to influence the summary statistics substantially. Third, publication bias seemed apparent in the combined analysis of all of the included studies and adjustment for this using the “trim-and-fill” method changed the summary estimate significantly. Nevertheless, no significant publication bias was detected in the subgroup analysis.

In conclusion, our meta-analysis showed that high SIRT1 expression was clearly associated with worse OS in non-colorectal gastrointestinal cancer, in particular in hepatocellular carcinoma and gastric cancer. Moreover, the current study supported the viewpoint that there is no correlation between SIRT1 expression and survival in colorectal cancer. However, this result should be interpreted with caution due to heterogeneity among colorectal studies. Further studies with large sample sizes and stratified analyses according to clinicopathological characteristics, as well as other colorectal cancer-related molecular biomarkers, are needed to evaluate the prognostic role of SIRT1 more precisely.

## MATERIALS AND METHODS

### Identification and selection of studies

PubMed was searched to identify studies evaluating the expression of SIRT1 and survival outcomes in cancer patients through March 2017. The search terms included “SIRT1” or “sirtuin 1” or “sir2”, combined with “survival” or “prognosis” or “outcome”, and combined with “cancer”. In addition, we used the name of each specific digestive system cancer (for example, colorectal cancer) instead of the search term “cancer” to recognize additional studies. Peer-reviewed studies were eligible and included if they met the following criteria: 1) studies included patients with gastrointestinal cancer; 2) studies explored the relationships between the tumoral expression of SIRT1 and OS; 3) there was sufficient survival data to extract or estimate the individual hazard ratio; and 4) studies were published in English. If the data sets were duplicated, we included only the most recent study.

### Data extraction

Two authors independently collected the following information of each eligible study: first author, year of publication, cancer type, number of patients, patient source, primary antibody used for the evaluation of SIRT1, proportion with high SIRT1 expression, follow-up time, survival outcome data, and variables adjusted for in the analyses. When more than one HR was provided, the most adjusted HR was collected.

### Quality assessment

Methodological quality was assessed following the REMARK guideline for reporting tumor-marker studies [41]. A scoring system was developed according to previous studies [42]. Briefly, a total of eighteen items that could be grouped into five major classifications, including study design, assay method, confounders, outcome, and analysis, were extracted for assessment. Each item was scored as 0 (no matched description), 1 (partly description), or 2 (complete description). Thus, the final quality score for each of the included studies ranged from 0 to 36, with higher scores reflecting better methodological quality.

### Statistical analysis

The association between SIRT1 expression and OS was presented as the HR, with a higher HR reflecting increased hazard of all-cause mortality for OS. The survival data for eligible studies were summarized by using the log HR. For studies that did not provide the numerical data for the estimation of summary statistics, the log HR was estimated based on Kaplan-Meier curves, as previously described [43]. The statistical heterogeneity was assessed by the chi-based *Q*-test and the *I*^2^ test. The data from individual studies were pooled to generate the summary log HR and variance according to the heterogeneity among studies (*I*^2^ < 50%: the fixed-effects model; *I*^2^ ≥ 50%: the random-effects model). In addition, a sensitivity analysis, in which one study was omitted at a time from the summary estimate, was conducted to assess whether individual studies significantly influenced the summary statistic. The publication bias was evaluated by creating funnel plots, and was estimated by Egger’s and Begg’s tests.

All statistical analyses were performed using STATA 10.1. *P* values were two-tailed, and *P* < 0.05 was considered statistically significant.

## Abbreviations

SIR2: silent information regulator 2
SIRT1: sirtuin 1
OS: overall survival
sHR: summary hazard ratio
CI: confidence intervals
*P*_h_: *P* value for heterogeneity

## References

[1] Ferlay J, Soerjomataram I, Dikshit R, Eser S, Mathers C, Rebelo M, Parkin DM, Forman D, Bray F (2015). Cancer incidence and mortality worldwide: sources, methods and major patterns in GLOBOCAN 2012. Int J Cancer.

[2] West AC, Johnstone RW (2014). New and emerging HDAC inhibitors for cancer treatment. J Clin Invest.

[3] Song NY, Surh YJ (2012). Janus-faced role of SIRT1 in tumorigenesis. Ann N Y Acad Sci.

[4] Chen WY, Wang DH, Yen RC, Luo J, Gu W, Baylin SB (2005). Tumor suppressor HIC1 directly regulates SIRT1 to modulate p53-dependent DNA-damage responses. Cell.

[5] Dai JM, Wang ZY, Sun DC, Lin RX, Wang SQ (2007). SIRT1 interacts with p73 and suppresses p73-dependent transcriptional activity. J Cell Physiol.

[6] Marshall GM, Liu PY, Gherardi S, Scarlett CJ, Bedalov A, Xu N, Iraci N, Valli E, Ling D, Thomas W, van Bekkum M, Sekyere E, Jankowski K (2011). SIRT1 promotes N-Myc oncogenesis through a positive feedback loop involving the effects of MKP3 and ERK on N-Myc protein stability. PLoS Genet.

[7] Ford J, Jiang M, Milner J (2005). Cancer-specific functions of SIRT1 enable human epithelial cancer cell growth and survival. Cancer Res.

[8] Herranz D, Maraver A, Canamero M, Gomez-Lopez G, Inglada-Perez L, Robledo M, Castelblanco E, Matias-Guiu X, Serrano M (2013). SIRT1 promotes thyroid carcinogenesis driven by PTEN deficiency. Oncogene.

[9] Leko V, Park GJ, Lao U, Simon JA, Bedalov A (2013). Enterocyte-specific inactivation of SIRT1 reduces tumor load in the APC(+/min) mouse model. PLoS One.

[10] Saunders LR, Verdin E (2007). Sirtuins: critical regulators at the crossroads between cancer and aging. Oncogene.

[11] Chen J, Zhang B, Wong N, Lo AW, To KF, Chan AW, Ng MH, Ho CY, Cheng SH, Lai PB, Yu J, Ng HK, Ling MT (2011). Sirtuin 1 is upregulated in a subset of hepatocellular carcinomas where it is essential for telomere maintenance and tumor cell growth. Cancer Res.

[12] Chen HC, Jeng YM, Yuan RH, Hsu HC, Chen YL (2012). SIRT1 promotes tumorigenesis and resistance to chemotherapy in hepatocellular carcinoma and its expression predicts poor prognosis. Ann Surg Oncol.

[13] Zhao G, Qin Q, Zhang J, Liu Y, Deng S, Liu L, Wang B, Tian K, Wang C (2013). Hypermethylation of HIC1 promoter and aberrant expression of HIC1/SIRT1 might contribute to the carcinogenesis of pancreatic cancer. Ann Surg Oncol.

[14] Zhao G, Cui J, Zhang JG, Qin Q, Chen Q, Yin T, Deng SC, Liu Y, Liu L, Wang B, Tian K, Wang GB, Wang CY (2011). SIRT1 RNAi knockdown induces apoptosis and senescence, inhibits invasion and enhances chemosensitivity in pancreatic cancer cells. Gene Ther.

[15] Lv L, Shen Z, Zhang J, Zhang H, Dong J, Yan Y, Liu F, Jiang K, Ye Y, Wang S (2014). Clinicopathological significance of SIRT1 expression in colorectal adenocarcinoma. Med Oncol.

[16] Chen X, Sun K, Jiao S, Cai N, Zhao X, Zou H, Xie Y, Wang Z, Zhong M, Wei L (2014). High levels of SIRT1 expression enhance tumorigenesis and associate with a poor prognosis of colorectal carcinoma patients. Sci Rep.

[17] Wauters E, Sanchez-Arevalo Lobo VJ, Pinho AV, Mawson A, Herranz D, Wu J, Cowley MJ, Colvin EK, Njicop EN, Sutherland RL, Liu T, Serrano M, Bouwens L (2013). Sirtuin-1 regulates acinar-to-ductal metaplasia and supports cancer cell viability in pancreatic cancer. Cancer Res.

[18] Hirai S, Endo S, Saito R, Hirose M, Ueno T, Suzuki H, Yamato K, Abei M, Hyodo I (2014). Antitumor effects of a sirtuin inhibitor, tenovin-6, against gastric cancer cells via death receptor 5 up-regulation. PLoS One.

[19] Ueno T, Endo S, Saito R, Hirose M, Hirai S, Suzuki H, Yamato K, Hyodo I (2013). The sirtuin inhibitor tenovin-6 upregulates death receptor 5 and enhances cytotoxic effects of 5-fluorouracil and oxaliplatin in colon cancer cells. Oncol Res.

[20] Zhang JG, Hong DF, Zhang CW, Sun XD, Wang ZF, Shi Y, Liu JW, Shen GL, Zhang YB, Cheng J, Wang CY, Zhao G (2014). Sirtuin 1 facilitates chemoresistance of pancreatic cancer cells by regulating adaptive response to chemotherapy-induced stress. Cancer Sci.

[21] Portmann S, Fahrner R, Lechleiter A, Keogh A, Overney S, Laemmle A, Mikami K, Montani M, Tschan MP, Candinas D, Stroka D (2013). Antitumor effect of SIRT1 inhibition in human HCC tumor models *in vitro* and *in vivo*. Mol Cancer Ther.

[22] Jang KY, Noh SJ, Lehwald N, Tao GZ, Bellovin DI, Park HS, Moon WS, Felsher DW, Sylvester KG (2012). SIRT1 and c-Myc promote liver tumor cell survival and predict poor survival of human hepatocellular carcinomas. PLoS One.

[23] Stenzinger A, Endris V, Klauschen F, Sinn B, Lorenz K, Warth A, Goeppert B, Ehemann V, Muckenhuber A, Kamphues C, Bahra M, Neuhaus P, Weichert W (2013). High SIRT1 expression is a negative prognosticator in pancreatic ductal adenocarcinoma. BMC Cancer.

[24] Cha EJ, Noh SJ, Kwon KS, Kim CY, Park BH, Park HS, Lee H, Chung MJ, Kang MJ, Lee DG, Moon WS, Jang KY (2009). Expression of DBC1 and SIRT1 is associated with poor prognosis of gastric carcinoma. Clin Cancer Res.

[25] Nosho K, Shima K, Irahara N, Kure S, Firestein R, Baba Y, Toyoda S, Chen L, Hazra A, Giovannucci EL, Fuchs CS, Ogino S (2009). SIRT1 histone deacetylase expression is associated with microsatellite instability and CpG island methylator phenotype in colorectal cancer. Mod Pathol.

[26] Hao C, Zhu PX, Yang X, Han ZP, Jiang JH, Zong C, Zhang XG, Liu WT, Zhao QD, Fan TT, Zhang L, Wei LX (2014). Overexpression of SIRT1 promotes metastasis through epithelial-mesenchymal transition in hepatocellular carcinoma. BMC Cancer.

[27] Benard A, Goossens-Beumer IJ, van Hoesel AQ, Horati H, de Graaf W, Putter H, Zeestraten EC, Liefers GJ, van de Velde CJ, Kuppen PJ (2015). Nuclear expression of histone deacetylases and their histone modifications predicts clinical outcome in colorectal cancer. Histopathology.

[28] Jung W, Hong KD, Jung WY, Lee E, Shin BK, Kim HK, Kim A, Kim BH (2013). SIRT1 expression is associated with good prognosis in colorectal cancer. Korean J Pathol.

[29] Jang SH, Min KW, Paik SS, Jang KS (2012). Loss of SIRT1 histone deacetylase expression associates with tumour progression in colorectal adenocarcinoma. J Clin Pathol.

[30] Qiu G, Li X, Wei C, Che X, He S, Lu J, Wang S, Pang K, Fan L (2016). The prognostic role of SIRT1-autophagy axis in gastric cancer. Dis Markers.

[31] Zhang S, Huang S, Deng C, Cao Y, Yang J, Chen G, Zhang B, Duan C, Shi J, Kong B, Friess H, Zhao N, Huang C (2017). Co-ordinated overexpression of SIRT1 and STAT3 is associated with poor survival outcome in gastric cancer patients. Oncotarget.

[32] He Z, Yi J, Jin L, Pan B, Chen L, Song H (2016). Overexpression of Sirtuin-1 is associated with poor clinical outcome in esophageal squamous cell carcinoma. Tumour Biol.

[33] Zhang LH, Huang Q, Fan XS, Wu HY, Yang J, Feng AN (2013). Clinicopathological significance of SIRT1 and p300/CBP expression in gastroesophageal junction (GEJ) cancer and the correlation with E-cadherin and MLH1. Pathol Res Pract.

[34] Liu T, Liu PY, Marshall GM (2009). The critical role of the class III histone deacetylase SIRT1 in cancer. Cancer Res.

[35] Zhu H, Xia L, Zhang Y, Wang H, Xu W, Hu H, Wang J, Xin J, Gang Y, Sha S, Xu B, Fan D, Nie Y (2012). Activating transcription factor 4 confers a multidrug resistance phenotype to gastric cancer cells through transactivation of SIRT1 expression. PLoS One.

[36] Zu G, Ji A, Zhou T, Che N (2016). Clinicopathological significance of SIRT1 expression in colorectal cancer: a systematic review and meta analysis. Int J Surg.

[37] Kabra N, Li Z, Chen L, Li B, Zhang X, Wang C, Yeatman T, Coppola D, Chen J (2009). SirT1 is an inhibitor of proliferation and tumor formation in colon cancer. J Biol Chem.

[38] Kriegl L, Vieth M, Kirchner T, Menssen A (2012). Up-regulation of c-MYC, SIRT1 expression correlates with malignant transformation in the serrated route to colorectal cancer. Oncotarget.

[39] Guastadisegni C, Colafranceschi M, Ottini L, Dogliotti E (2010). Microsatellite instability as a marker of prognosis and response to therapy: a meta-analysis of colorectal cancer survival data. Eur J Cancer.

[40] Juo YY, Johnston FM, Zhang DY, Juo HH, Wang H, Pappou EP, Yu T, Easwaran H, Baylin S, van Engeland M, Ahuja N (2014). Prognostic value of CpG island methylator phenotype among colorectal cancer patients: a systematic review and meta-analysis. Ann Oncol.

[41] McShane LM, Altman DG, Sauerbrei W, Taube SE, Gion M, Clark GM (2005). Statistics Subcommittee of the NCIEWGoCD. REporting recommendations for tumour MARKer prognostic studies (REMARK). Br J Cancer.

[42] Sun DW, Zhang YY, Qi Y, Zhou XT, Lv GY (2015). Prognostic significance of MMP-7 expression in colorectal cancer: a meta-analysis. Cancer Epidemiol.

[43] Tierney JF, Stewart LA, Ghersi D, Burdett S, Sydes MR (2007). Practical methods for incorporating summary time-to-event data into meta-analysis. Trials.

